# SOCIAL DISPARITIES IN SLEEP PATTERNS AMONG US ADULTS IN MID AND OLDER ADULTHOOD

**DOI:** 10.1093/geroni/igad104.0665

**Published:** 2023-12-21

**Authors:** Connor Sheehan

**Affiliations:** Arizona State University, Tempe, Arizona, United States

## Abstract

As the U.S. population ages, understanding social disparities in sleep among older adults is paramount. Even as humans physiologically require sleep, sleep duration decreases, and insomnia increases throughout older adulthood. Further, the implications of sleep related conditions (e.g., heart disease) are elevated in older adulthood. Importantly, these changes in sleep patterns throughout older adulthood are distributed in a manner that reflects broader social inequities, as quality sleep is contingent upon resources that are distributed unequally across society (e.g., time, stress, quiet, comfort, access to quality medical care). The inequities in sleep have profound implications for intergenerational social inequality, well-being, and longevity. Novel results regarding social based sleep disparities by gender, race/ethnicity, and educational attainment among older adults from the Health and Retirement Study and the National Health Interview Survey will be presented. The profound implications of sleep disparities and potential interventions will also be discussed.

